# Development of Microstructural and Morphological Cortical Profiles in the Neonatal Brain

**DOI:** 10.1093/cercor/bhaa150

**Published:** 2020-06-12

**Authors:** Daphna Fenchel, Ralica Dimitrova, Jakob Seidlitz, Emma C Robinson, Dafnis Batalle, Jana Hutter, Daan Christiaens, Maximilian Pietsch, Jakki Brandon, Emer J Hughes, Joanna Allsop, Camilla O’Keeffe, Anthony N Price, Lucilio Cordero-Grande, Andreas Schuh, Antonios Makropoulos, Jonathan Passerat-Palmbach, Jelena Bozek, Daniel Rueckert, Joseph V Hajnal, Armin Raznahan, Grainne McAlonan, A David Edwards, Jonathan O’Muircheartaigh

**Affiliations:** 1 MRC Centre for Neurodevelopmental Disorders, King’s College London, London, SE1 1UL, UK; 2 Sackler Institute for Translational Neurodevelopment, Institute of Psychiatry, Psychology & Neuroscience, King’s College London, London, SE5 8AF, UK; 3 Department of Forensic and Neurodevelopmental Sciences, Institute of Psychiatry, Psychology & Neuroscience, King's College London, London, SE5 8AF, UK; 4 Department of Perinatal Imaging & Health, School of Biomedical Engineering & Imaging Sciences, Centre for the Developing Brain, King's College London, London, SE1 7EH, UK; 5 Developmental Neurogenomics Unit, National Institute of Mental Health, Bethesda, MD 20892, USA; 6 Department of Psychiatry, University of Cambridge, Cambridge, CB2 0SZ, UK; 7 Department of Biomedical Engineering, School of Biomedical Engineering & Imaging Sciences, King's College London, London, SE1 7EU, UK; 8 Biomedical Image Analysis Group, Department of Computing, Imperial College London, London, SW7 2AZ, UK; 9 Faculty of Electrical Engineering and Computing, University of Zagreb, Zagreb, 10000, Croatia; 10 South London and Maudsley NHS Foundation Trust, London, SE5 8AZ, UK

**Keywords:** developing brain, morphometric similarity networks, multimodal MRI, perinatal, structural covariance

## Abstract

Interruptions to neurodevelopment during the perinatal period may have long-lasting consequences. However, to be able to investigate deviations in the foundation of proper connectivity and functional circuits, we need a measure of how this architecture evolves in the typically developing brain. To this end, in a cohort of 241 term-born infants, we used magnetic resonance imaging to estimate cortical profiles based on morphometry and microstructure over the perinatal period (37–44 weeks postmenstrual age, PMA). Using the covariance of these profiles as a measure of inter-areal network similarity (morphometric similarity networks; MSN), we clustered these networks into distinct modules. The resulting modules were consistent and symmetric, and corresponded to known functional distinctions, including sensory–motor, limbic, and association regions, and were spatially mapped onto known cytoarchitectonic tissue classes. Posterior regions became more morphometrically similar with increasing age, while peri-cingulate and medial temporal regions became more dissimilar. Network strength was associated with age: Within-network similarity increased over age suggesting emerging network distinction. These changes in cortical network architecture over an 8-week period are consistent with, and likely underpin, the highly dynamic processes occurring during this critical period. The resulting cortical profiles might provide normative reference to investigate atypical early brain development.

## Introduction

Brain maturation over the perinatal period is rapid and complex. Although the majority of neurons are at their terminal location, synaptogenesis and myelination are ongoing, and (limited) migration of interneurons continues (Petanjek et al. 2011; Paredes et al. 2016). In the cortex, these processes are coordinated with sensory cortex and pathways developing earliest, prefrontal and association areas later (Flechsig of Leipsic 1901; Huttenlocher 1979). This developing architecture and connectivity is critical for efficient functional signaling, supporting and enabling the later development of cognitive and behavioral abilities.

Although alterations in perinatal neurodevelopmental processes have been associated with later cognitive and behavioral difficulties, quantifying myelo- or cytoarchitecture anatomical circuit development in the living human neonate is challenging. A relatively simple hypothesis of cortical connectivity is “similar prefers similar” (Goulas et al. 2016), meaning areas with similar cytoarchitecture preferentially connect. In this context, magnetic resonance imaging (MRI)-based methods such as regional structural covariance (Evans 2013) offer a proxy measure of brain connectivity, with structural similarity of spatially distinct regions of cortex reflecting coordinated maturation (Alexander-Bloch et al. 2013a). This inter-regional similarity is supported by similar genetic or maturational profiles (Alexander-Bloch et al. 2013b) and transcriptomic profiles (Yee et al. 2018) or associated with changes in disease (e.g., Zuo et al. 2018).

Understanding early structural regional similarity can shed light on how the brain develops into an efficient multifunctional system (Cao et al. 2016) and allows the detection of perturbations in normal development (Di Martino et al. 2014; Morgan et al. 2018). Recent years have seen a rise in studies on brain connectivity networks in the perinatal period (Zhao et al. 2019). Structural covariance networks (SCNs) based on measures of gray matter (GM) volume, cortical thickness (CT), cortical folding, and fiber density in the first 2 years of life have been described (Fan et al. 2011; Nie et al. 2014; Geng et al. 2017). Graph measures of these networks at birth show similar organizational properties to brain networks described in adulthood, suggesting that the nonrandom, efficient architecture of the brain is an inherent characteristic of the system, evident from very early on (Fan et al. 2011; Shi et al. 2012). Moreover, measures derived from GM volume covariance networks were found to differ in neonates with familial risk of schizophrenia (Shi et al. 2012). As different anatomical measures are capturing distinct developmental processes (Rakic 1988) and are controlled by different genetic mechanisms (Panizzon et al. 2009; Chen et al. 2013), different measures of cortical development show specific spatial and temporal patterns in early childhood (Gilmore et al. 2012; Li et al. 2013; Nie et al. 2014; Lyall et al. 2015). Therefore, as might be expected, the resulting single-feature SCNs can be inconsistent.

An alternative approach using multiple anatomical measures to elucidate regional structural similarity has recently been examined in adolescents and adults (Li et al. 2017; Seidlitz et al. 2018). Compared with SCNs derived from a single technique, this approach allows for the construction of networks for individuals, rather than over a group, enabling the assessment of individual variability masked by group templates or case-control studies (Seghier and Price 2018). The resulting morphometric similarity networks (MSNs) have superior spatial consistency with cortical cytoarchitecture compared with networks based on diffusion imaging or on one structural measure (CT) (Seidlitz et al. 2018). Furthermore, cortical regions shown to be connected (i.e., similar) based on this approach have complementary expression of specific genes (Romero-Garcia et al. 2018; Seidlitz et al. 2018). In adults, these networks are also sensitive to alterations in common (Morgan et al. 2019) and rare (Seidlitz et al. 2019) neurodevelopmental disorders. They also have neurobiological specificity; cortical MSNs differ between patients with genetic syndromes and are aligned with the regional expression of disorder-related genes (Seidlitz et al. 2019). The only work we found to apply MSNs in the perinatal period is a recent study in neonates using a variant of MSNs, where authors were able to successfully predict postmenstrual age (PMA) at scan and differentiated infants born prematurely, with superior performance compared with using single predictive measures (Galdi et al. 2020).

The aim of this work therefore was to examine cortical organization from a multi-morphometric perspective, utilizing MSNs; hypothesizing this approach will provide a more sensitive and informative means to describe the postnatal structure and cytoarchitecture of the brain. We constructed cortical MSNs based on structural/morphological and microstructural diffusion parameters in a sample of 241 infant scanned between 37 and 44 weeks PMA. We hypothesized that, in the period following birth, brain development will be reflected in these networks. Moreover, we were interested in the community structure, or modularity in the neonatal brain, assuming these early clusters will provide the skeleton for developing functionality.

## Materials and Methods

### Subjects

This work included a sample of neonates participating in the Developing Human Connectome Project (dHCP) (http://www.developingconnectome.org/), scanned at the Newborn Imaging Centre at Evelina London Children’s Hospital, London, UK. Images are available for download and analysis at the project website. This project has received ethical approval (14/LO/1169, IRAS 138070), and written informed consent was obtained from parents. At the beginning of this specific analysis (October 2018), 383 singleton term-born babies had undergone successfully structural and diffusion acquisitions, reconstruction, and early preprocessing. We next removed 87 subjects who failed the structural or diffusion pipelines as described below or who had missing data (e.g., T1 image). Finally, we removed subjects that have not passed diffusion QC: 17 subjects at the lower 5% of motion parameters and 46 subjects whose brains moved out of the field of view during scanning, leading to slices missing on the superior surface of the brain. One subject with major basal ganglia lesion was excluded, resulting in a final sample of 241 subjects (born at between 37 and 42 weeks; 128 males) with MR images acquired at PMA 40.92 ± 1.58 weeks (mean ± sd), range 37.43–44.71 weeks, henceforth “age.” No major brain abnormalities were detected in review of the MRI data by a neonatal neuroradiologist in the subjects included.

### Image Acquisition

MR images were acquired on a 3T Philips Achieva scanner without sedation, using a dedicated 32-channel neonatal head coil system (Hughes et al. 2017). Acquisition and reconstruction followed optimized protocols for structural images (Kuklisova-Murgasova et al. 2012; Cordero-Grande et al. 2016, 2018) and multi-shell high angular resolution diffusion imaging (HARDI) (Tournier et al. 2015; Hutter et al. 2018). T2-weighted (T2w) images were obtained using a turbo spin echo (TSE) sequence, acquired in two stacks of 2D slices (in sagittal and axial planes), using parameters: TR = 12 s, TE = 156 ms, and SENSE factor 2.11 (axial) and 2.58 (sagittal) with overlapping slices (resolution 0.8 × 0.8 × 1.6 mm). T1-weighted (T1w) images were acquired using an IR (inversion recovery) TSE sequence with the same resolution using TR = 4.8 s, TE = 8.7 ms, and SENSE factor 2.26 (axial) and 2.66 (sagittal). Structural images were reconstructed to a final resolution 0.5 × 0.5 × 0.5 mm, using slice-to-volume registration. Diffusion images were obtained using parameters TR = 3800 ms, TE = 90 ms, SENSE factor = 1.2, multiband factor = 4, and resolution 1.5 × 1.5 × 3.0 mm with 1.5 mm slice overlap. Diffusion gradient encoding included images collected at *b* = 0 s/mm^2^ (20 repeats), *b* = 400 s/mm^2^ (64 directions), *b* = 1000s/mm^2^ (88 directions), and *b* = 2600 s/mm^2^ (128 directions), and images were reconstructed to a final resolution of 1.5 × 1.5 × 1.5 mm.

### Image Processing

Cortical surface processing and extraction of individual cortical features followed the pipeline described in Makropoulos et al. (2018)). Briefly, motion- and bias-corrected T2w images were brain extracted and segmented. White, pial, and midthickness surfaces were extracted, inflated, and projected onto a sphere. This was followed by estimation of cortical features including CT, pial surface area (SA), mean curvature (MC), and a gross proxy of myelin content, defined as the ratio between the T1w and T2w images (Glasser and Van Essen 2011), performed after registering the individual T1 and T2 images together, henceforth “myelin index (MI),” as detailed in Makropoulos et al. (2018). All brains were aligned to the 40-week dHCP surface template (Bozek et al. 2018) using Multimodal Surface Matching (MSM) (Robinson et al. 2013, 2014), run with higher-order regularization constraints (Robinson et al. 2018), to match coarse scale cortical folding (sulcal depth) maps. All other metrics were resampled to the template using this transformation and adaptive barycentric resampling (implemented using Human Connectome Project (HCP) tools, Connectome Workbench (https://www.humanconnectome.org/software/connectome-workbench).

Diffusion images were denoised (Veraart et al. 2016), Gibbs-ringing suppressed (Kellner et al. 2016), and corrected for subject motion and image distortion with slice-to-volume reconstruction in the multi-shell spherical harmonics and radial decomposition (SHARD) basis (Christiaens et al. 2019a), as described in Christiaens et al. (2019b) and using an image-based field map (Andersson et al. 2003). A tensor model for diffusion data was then fitted using a single shell (*b* = 1000), and fractional anisotropy (FA) and mean diffusivity (MD) maps were generated using MRtrix3 (Tournier et al. 2019). Neurite density index (NDI) and orientation dispersion index (ODI) maps were calculated using the NODDI toolbox (Zhang et al. 2012) as previously applied in the neonatal brain (Batalle et al. 2019). We acknowledge that the default parameters used to model NODDI here may not be the optimal for our sample (Guerrero et al. 2019). However, there is currently no agreed standard for infant-specific parameters; therefore we opted for the default to at least permit comparisons with previous literature.

Diffusion maps were registered onto individual T2w images using FSL’s epi_reg (FLIRT) (https://fsl.fmrib.ox.ac.uk) and then projected onto cortical surface using Connectome Workbench in order to sample imaging features with the same spatial representation. All raw images were visually inspected for motion or image artifact and artifacted data excluded, and processed images were inspected for registration errors.

### MSNs Construction

**Figure 1:**
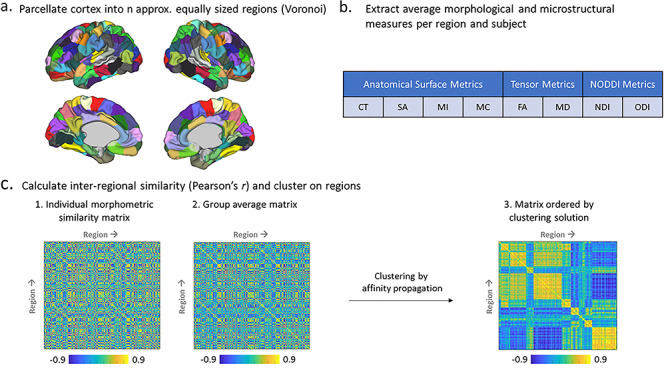
Pipeline for clustering MSNs in the neonatal brain: (*a*) Cortical regions are defined using Voronoi tessellation of the cortical surface. (*b*) Each individual region is characterized by an eight-feature vector including averaged normalized values of cortical thickness (CT), mean curvature (MC), myelin index (MI), surface area (SA), fractional anisotropy (FA), mean diffusivity (MD), neurite density index (NDI), and orientation dispersion index (ODI). (*c*) Each pair of regions is correlated using Pearson’s *r*, resulting in a subject-specific similarity matrix with size *n* regions × *n* regions, which are then averaged to create a group mean similarity matrix, and the resulting group matrix is clustered using affinity propagation algorithm to examine network modularity.

Cortical regions were defined using approximately equal-sized cortical parcellations with Voronoi decomposition at different granularities (*n* = 50, 100, 150, 200, 250, 300). This option was chosen over parcellation with a pre-existing atlas in order to minimize the effect of variable regional size when calculating the MSNs. For clarity and presentation, all results shown (other than Supplementary Fig. 1) were based on the *n* = 150 parcellation. Network construction followed steps introduced before (Li et al. 2017; Seidlitz et al. 2018): For each subject, each of the eight features was first *z*-scored across the cortex (to account for feature variation) and then averaged across each ROI in the parcellation, resulting in an eight-feature vector including mean normalized values of CT, MC, MI, SA, FA, MD, NDI, and ODI characterizing each node. Correlation (Pearson’s *r*) between the eight-feature vectors for every two pairs of regions was calculated using Matlab, resulting in an *n* regions *× n* regions similarity matrix for each subject. A group structural similarity matrix was produced by averaging the 241 individual similarity matrices (Fig. 1), and clustering was performed with affinity propagation. This process was also applied for a “leave-one-out” analysis, where similarity was based on a series of seven-feature vectors, leaving one feature out each time. In addition, typical SCNs for the individual features which are much more common in the literature were also generated by correlating each pair of regions across all subjects, resulting in a group similarity matrix that is based on a single morphometric feature. The values used in this analysis were the raw, un-normalized values.

### Association Between MSNs with Age at Scan and Sex

To illustrate the effect of age on the individual morphometric features, Spearman’s correlation (Spearman’s rho, $ \rho $) between the averaged regional single features and age was calculated and results corrected with false discovery rate (FDR) at 5% (Benjamini and Hochberg 1995). Nonparametric correlations were chosen for all age analyses as age at scan was not normally distributed in this sample as indicated by Shapiro–Wilk test (W(241) = 0.98, *P* = 0.02).

The effects of age on internodal similarity were examined by correlations between age and the internodal edge-strength. In addition, we investigated the same correlation with *mean* node strength (the *average* of a node’s edge-strength with every other node). Sex differences in MSNs for individual edge-strength and mean nodal edge-strength were explored using the Mann–Whitney test. In these contrasts, results were corrected with pFDR (Storey 2002; Storey and Tibshirani 2003), which is advantageous over FDR in larger samples.

### MSN Clustering Analysis

To investigate network modularity, the group similarity matrix was clustered using affinity propagation (Frey and Dueck 2007, https://psi.toronto.edu/?q=tools). This clustering method has two merits in this current work: First, clustering could be performed without thresholding the group matrix, therefore avoiding information loss due to binarization, and second, it returns the optimal number of clusters (*k*) for a given preference value. In our case we used the median of the similarity matrix as preference value, making no prior assumptions: While a prespecified *k* is not mandatory, the algorithm does require an input chosen by the user, termed “preference value.” By choosing the median of the similarity matrix as a shared value, we instruct the algorithm to consider all data points or in our case nodes as “exemplars” for the clusters. In addition, we also performed the same clustering approach but restricted the solution to a *k* = 7, the number of tissue classes in our von Economo atlas (see below), allowing us to compare this (fixed) clustering solution to clustering solutions from other modalities (see below). The rationale behind *k* = 7 clusters is also based on the maximum rank of the data in the context of a PCA, where the maximum number of components out of eight features from which the MSN was derived (as is the case here) is *k* − 1.

To measure robustness of the MSN clusters to the type of MRI modalities included, and to rank the importance of input modalities to the eventual solution, agreement between the clustering solution for the eight-feature regional vectors and the clustering solutions for seven-feature regional vectors (leave-one-out analysis) and clustering solutions for the eight single features (i.e., SCNs) was examined using normalized variation of information (Meilă 2007; Reichart and Rappoport 2009) using the ‘nvi’ code from the Pattern Recognition and Machine Learning Toolbox for Matlab (http://prml.github.io/). As in the leave-one-feature-out analysis, we fixed *k* = 7. In order to rule out the possibility that the clustering solution is derived only from age effects, we also performed clustering on the correlation between the edge-strength and age.

As MSNs in adults have been reported to correspond with cortical cytoarchitecture (Seidlitz et al. 2018), we sought to examine the overlap between the resulting clusters with gross von Economo cytoarchitectural classifications (seven classes) (von Economo and Koskinas 1925) using the Dice coefficient to estimate overlap as in Whitaker et al. (2016). We also attempted to examine the spatial overlap between the seven clusters and von Economo classes using a more rigorous method: spatial permutations of the spherical representation of the cortical surface (Alexander-Bloch et al. 2018). We generated 500 null distributions for assessing correspondence between the maps as measured by normalized variation of information, while correcting for multiple comparisons as described in Alexander-Bloch et al. (2018).

#### Association Between MSN Clusters and Age at Scan

To investigate developing network integration and segregation, for each cluster the averaged edge-strength of connections within a cluster (with higher values indicating cluster distinction within the entire network) and the averaged edge-strength of connections between nodes within the cluster and nodes external to that cluster (indicating integration or segregation of that cluster) were calculated. Spearman’s $ \rho $ coefficients were calculated for changes in these modular integration and segregation measures against age. Results were FDR corrected at 5% (Benjamini and Hochberg 1995).

## Results

### Age at Scan and Sex Effects

#### Age-Related Associations with Single-Feature Maps

**Figure 2:**
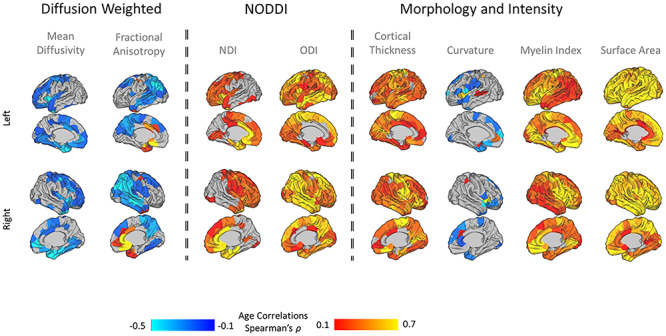
Association between single-feature maps (*n* = 150 parcels) and age at scan: Positive correlations are marked in red and negative correlations in blue. NDI, neurite density index; ODI, orientation dispersion index.

The correlation between parcellated single-feature maps and age at scan revealed metric-specific age associations: SA and MI had a brain-wide positive association with age, followed by CT, ODI, and NDI that also showed a positive association, but to a lesser extent. FA exhibited both positive and negative associations with age, while MD displayed only a negative association. Significant correlations with MC results were sparser, seen in limited perisylvian, frontal, and temporal regions (Fig. 2). Results are overlaid on a 41-week-old neonatal template (Bozek et al. 2018).

#### Age- and Sex-Related Associations with MSNs

**Figure 3:**
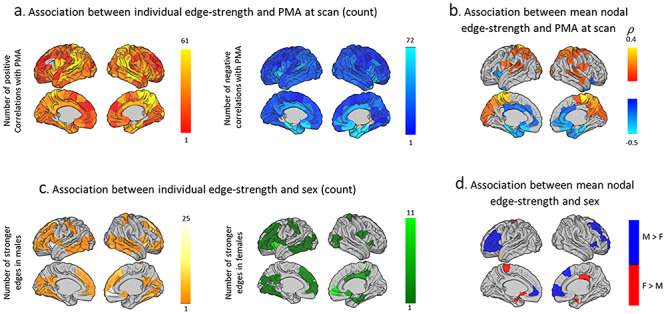
Age and sex associations with MSNs: (*a*) Sum of positive and negative correlations between internode edge-strength and age at scan. (*b*) Spearman’s correlation between age and mean nodal edge-strength. (*c*) Sum of stronger internode edges in males (orange) and in females (green). (*d*) Significant sex differences between mean nodal edge-strength.

The correlation between inter-regional edge-strength of MSNs (*n* = 150 parcels) and age at scan showed both positive and negative associations throughout the brain. They numbered less in frontal and anterior temporal regions (see Fig. 3*a* for a node-by-node count of significant edges). For mean nodal edge-strength, this spatial gradient was clearer, with anterior cingulate and limbic regions negatively associated with age and lateral and medial parietal positively associated (Fig. 3*b*). Individual edge-strength did show some sex associations (Fig. 3*c*), though less extensive, with higher edge-strength in lateral frontal and temporal regions in males and cingulum and medial temporal regions in females (Fig. 3*d*). Age at birth (*U* = 6910.5, *P* = 0.55) and at scan (*U* = 6817, *P* = 0.44) did not differ between males and females.

### Modularity of Neonatal MSNs

**Figure 4:**
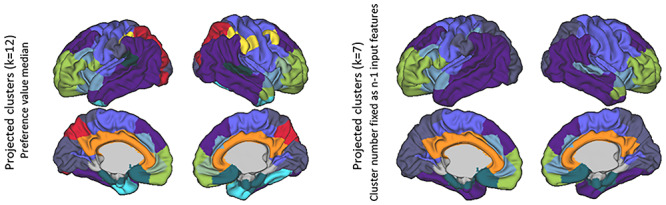
Clustering solution for MSNs created using eight features: Affinity propagation clustering based on a fixed preference value (median similarity) is shown on the left and for a fixed number of clusters on the right.

Using affinity propagation to perform clustering resulted in 12 broadly symmetric cortical modules, aligned with sensory–motor, fronto–temporal, anterior frontal, limbic, cingulate, insular, and visual systems (Fig. 4 left). Comparable spatial findings were also found when the initial parcellation included different number of regions (*n* = 50, 100, 150, 200, 250, 300), but the number of clusters increased linearly with parcellation density. For clarity, results are presented only for the middle density (*n* = 150).

A similar spatial partition was observed when the number of clusters was fixed at seven (Fig. 4 right) and across parcellation densities (Supplementary Fig. 1). The main difference between the 7- and 12-cluster solution was a subdivision of the fronto–temporal and occipital and parietal clusters in the *k* = 12 solution. Therefore, as these changes were minor, with no major loss of information on the overall emerging picture, and as a middle ground between complexity and interpretability, further analyses were performed on the *k* = 7 cluster solution.

To confirm the stability of this solution, clustering was calculated using bootstrapping of 20 random subjects each time, for 500 iterations, and individually for each subject. Node coincidence (how often one node fell in the same node as another) was very high (Supplementary Fig. 2*b*), and even in MSNs of individual infants, the consistency was very good (Supplementary Fig. 2*a*) with the whole sample cluster structure clearly evident in individuals and bootstrap samples.

**Figure 5:**
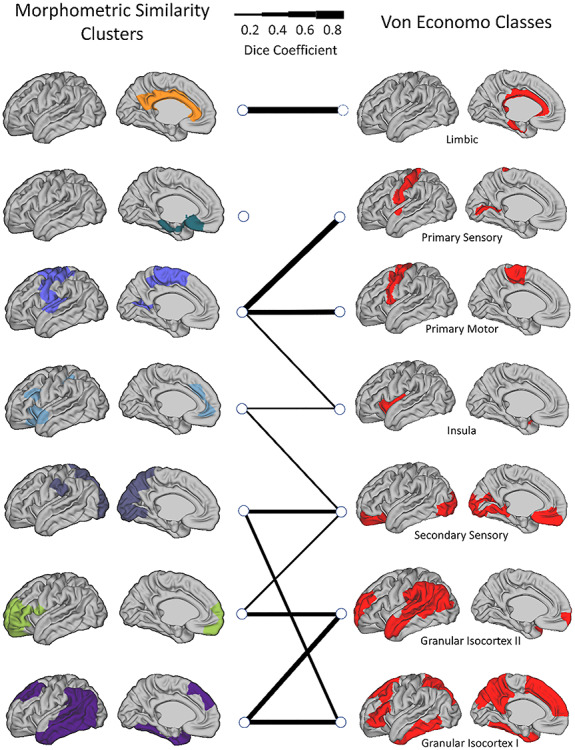
The spatial overlap (Dice coefficients) between MSN clusters and von Economo tissue classes: Line thickness indicates the Dice coefficient per pair of related regions and a Dice of <0.2 is not shown.

Comparing the resulting MSNs (for *k* = 7) and von Economo cytoarchitectural classes, similarities were found, more remarkably in limbic and primary sensory areas, as compared with association areas (Fig. 5). Using the spatial permutation test, the normalized variation of information, between the MSN clusters and von Economo classes, was 0.85, ranging *z* = 0.84–0.93, suggesting relativity modest overlap; however this overlap was more significant than would be expected by chance (*P* = 0.02).

#### Feature Contribution

**Figure 6:**
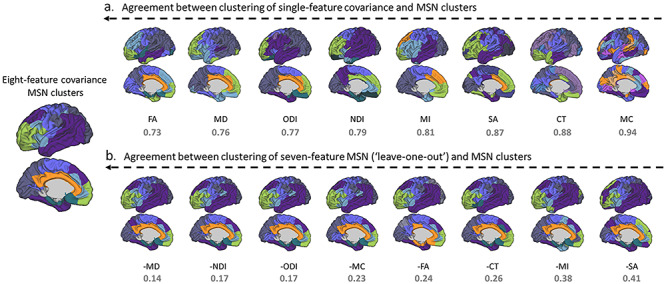
Agreement between clustering solutions using all features with single- and multimeasure covariance: results shown for left hemisphere for ease of interpretation. Clustering solutions are ordered from left (most similar) to right (least similar). (*a*) Level of agreement between modules derived from a single-feature covariance matrix and the eight-feature MSN. (*b*) Level of agreement between clustering solution for seven-feature MSN (leave-one-out analysis) and eight-feature MSN. In all cases the normalized variation of information value (*z*) is shown below (lower is better). CT, cortical thickness; MC, mean curvature; MI, myelin index; SA, surface area; FA, fractional anisotropy; MD, mean diffusivity; NDI, neurite density index; ODI, orientation dispersion index.

Examination of the clustering solution (for *k* = 7) for each of the *single* morphometric measures demonstrated that clustering of FA covariance had the highest agreement with the eight-feature solution (normalized variation of information, *z* = 0.73), while clustering of MC covariance had the lowest agreement (*z* = 0.94) (Fig. 6*a*). The top four metrics in particular are strongly correlated with each other cross-sectionally, derived from the same base diffusion-weighted images, and therefore this may indicate redundancy. This pattern remained when the single-feature SCNs were calculated with partial correlation, taking into account age, suggesting this is not exclusively derived by age effects in the individual modalities (data not shown).

In the “leave-one-out” seven-feature solutions, the agreement between modules was much higher than for single modalities, as might be expected. Removing SA and MI from MSN estimation had the most profound effect (e.g., resulted in more different clusters), indicating their higher importance to the eventual solution. Removing either MD, NDI, or ODI had less of an effect, with near identical solutions (Fig. 6*b*).

#### MSN Clusters and Age at Scan

**Figure 7:**
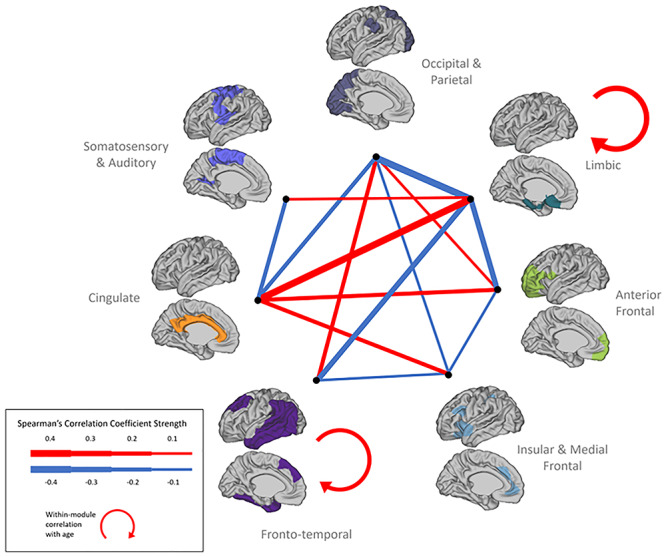
Inter- and intramodular similarity changes with age at scan: association between clusters’ edge-strength and age. Width of line is indicative of the strength of significant association.

To demonstrate the age-related changes in cluster differentiation over time, we performed correlations between individual measures of within-module similarity and between-module similarity. Between-module analysis revealed six cluster pairs with significant positive correlation with age, showing between-cluster integration over time—occipital–parietal and anterior frontal, occipital–parietal and fronto–temporal, limbic and somatosensory–auditory, limbic and cingulate, anterior–frontal and cingulate, and insular–medial frontal and cingulate—while eight cluster pairs showed significant negative correlation with age. The limbic and fronto–temporal clusters demonstrated increased within-module similarity with age, suggesting cluster distinction, or increased internal similarity, while no within-module negative correlation with age was found (Fig. 7). Exact statistical values appear in Supplementary Table 1.

#### Age-Related Associations with Clustering Solution

Given the clear association between age at scan and connection strength between edges and clusters, we investigated the resulting MSN modules when calculated on edge-by-edge age trajectories. We performed clustering on the correlation matrix of each node-to-node MSN edge against age. This solution demonstrated both a clearly different result (Supplementary Fig. 3) and also has a different interpretation, showing areas that are similar in their direction and strength of maturation. These results showed more long-range connections and much more heterogeneity in frontal cortex. Primary sensory regions clustered together, and bilateral symmetric fronto–temporal and fronto–parietal networks were also evident.

## Discussion

Cortical microstructure and morphology go through extensive changes postnatally. Though the majority of neurons are in situ at term age, myelination and synaptogenesis continue to refine the cortical architecture that will persist throughout the lifespan. Using a densely sampled dataset across the perinatal period, we were able for the first time to combine multiple structural imaging features and showed their utility in exploring the dynamic progression of brain maturation. We demonstrated a clear and consistent modular structure, with both local and distributed networks of brain regions having similar cortical profiles. This modular structure showed hemispheric symmetry, with correspondence to known long-range cortical functional networks. This structure partly mirrored von Economo classes, indicative of an underlying cellular composition upon which specialized cortical systems will emerge. To our knowledge, this is the first study in neonates using a multiparametric approach to examine the community structure of the neonatal brain.

MSNs are able to capture the extensive changes occurring throughout a short period of just 8 weeks following birth. Age had a complex effect on edge-strength showing both positive and negative correlations, i.e., regional profiles became more and less similar with age. These age associations were confined to occipital, parietal, and temporal areas and were less evident in frontal areas. Whereas these associations were positive (greater morphometric similarity with the rest of the brain), limbic and anterior cingulate regions showed negative correlations with age (less similarity). These findings suggest that in the neonatal period, sensory and limbic areas and posterior parietal regions have the largest maturational changes, as opposed to regions related to higher executive functions (e.g., prefrontal cortex), qualitatively similar to the pattern seen in Lebenberg et al. (2019) with older infants. Changes may only be evident in higher-order areas at a later stage, in line with the more prolonged synaptogenesis, dendritic arborization, and myelination patterns (Huttenlocher 1990; Huttenlocher and Dabholkar 1997; Teffer and Semendeferi 2012). This may also reflect work in cortical GM volume maturation in neonates, showing a posterior–anterior gradient in the first weeks following birth (Gilmore et al. 2007). In all, age-dependent changes observed in MSNs in the newborn are in line with neurobiological findings, as well as other neuroimaging findings.

In a mixed sample of term and preterm infants investigating MSNs (derived from structural and extended diffusion metrics, but not surface measures), Galdi et al. (2020) also found that brain structures became both more similar and dissimilar to each other over the same age range. While that study used different feature vectors, atlas, and populations here, findings are clearly complementary: Occipital connections increased with age, parietal edges were also more positively than negatively associated with age, while temporal edges showed mixed associations with age. In contrast to our findings, frontal regions also showed a strong relationship with age; however, this was more evident in white matter (WM) rather than GM. By choosing to focus only on healthy term-born infants, we attempted to minimize any effects of preterm status confounders on brain maturation (Ball et al. 2017; Batalle et al. 2017).

By exploring the developmental patterns of the individual metrics on which cortical covariance was based, as well as their contribution to the resulting cortical patterning, we were able to dissect early micro and macro properties of the brain. When examining each morphometric measure in isolation, unique developmental trajectories emerged, replicating prior work (Dean et al. 2017; Batalle et al. 2019). However, we also show that no one measure was able to capture the abundance of unique information that is reflected in the combination of morphometric features. Typical measures of cortical anatomy, thickness, and area, as well as myelin content, showed strong increase with age (Li et al. 2013). Age however showed little significant association with curvature, in agreement with previous reports showing relatively little change in MC compared with other surface measures in the months following birth (Li et al. 2015; Batalle et al. 2019), suggesting that cortical folding remains relatively static in this period. Though no single individual measure mirrored the clustering solution achieved by combining all measures, the diffusion metrics had the better correspondence (Fig. 6*a*) but were apparently less important to the clustering solution (Fig. 6*b*). This apparent contradiction is due to redundancy. For example, MD and NDI show very similar patterns of correlation with age (Fig. 2), so removal of one or two diffusion measures does not reduce the effective information used to calculate correlations in MSNs.

Cortical diffusion metrics show complex maturational trajectories around term birth. Ball et al. (2013) and Batalle et al. (2019) have shown that MD decreases linearly with age, while FA decreases rapidly until week 38 PMA but then begins to slowly increase. This might explain why at the age range examined here (37–44 weeks) we observed both negative and positive associations between FA and age. Moreover, Batalle et al. found that NDI is positively associated with age, similar to the results here. ODI seemed to stabilize after 38 weeks, whereas we still observe a strong association with age after 38 weeks. We might hypothesize that this is related to the focus on preterm neonates in that work, where microstructural trajectories could differ due to earlier birth and the very different early environmental exposures that entail (both clinical and ex utero related).

Sex differences were less widespread compared with age effects. While sex differences in cortical volumes are evident from birth (Gilmore et al. 2007), they are subtle when looking at single modality covariance networks in early development (Zielinski et al. 2010; Geng et al. 2017). Using the MSNs approach, we were able to detect sex effects in some cortical areas, most pronounced in frontal and temporal regions. This corresponds to results of a neonatal study using tensor-based morphometry (Knickmeyer et al. 2014), and even in adults, multimodal imaging sex differences were mainly localized in the frontal lobe, followed by parietal and temporal lobes (Feis et al. 2013), so this may represent a consistent gross pattern throughout the lifespan.

Generally, any network construction and follow-up clustering analyses are highly reliant on several factors, including population and age range examined (Seidlitz et al. 2018; Morgan et al. 2019), type of morphometric measures used (Nie et al. 2013), parcellation (Arslan et al. 2018), threshold (Bordier et al. 2017), wiring cost (Betzel et al. 2017), and type of modularity estimation (Sporns and Betzel 2016) and are therefore likely to vary accordingly. For module definition, we applied affinity propagation to discover meaningful cortical clusters, without eliminating or changing any connections for network construction. In young adults, utilizing MSNs and the Louvain algorithm, Seidlitz et al. (2018) reported four cortical modules consistent with lobular division. Using affinity propagation to cluster similar developing regions in terms of CT and curvedness between the ages 3 and 20 years, Nie et al. (2013) found eight and five clusters, respectively, also with some alignment to lobular division, and indeed our parcellation based just on CT was qualitatively similar.

While most of the resulting modules presented in these studies tend to be spatially contiguous and local, our clustering solution showed also long-range connections and might be more indicative of possible functional and structural connectivity. The modules were broadly aligned with known functional systems and were relatively stable: These included sensory–motor, fronto–parietal, temporal, limbic, cingulate, and visual regions. Our correspondence between MSNs and functional systems may reflect the underlying architecture for later developed functional networks (van den Heuvel et al. 2015; Geng et al. 2017). Our resulting MSN clusters show some overlap to the von Economo tissue classifications, implying the possible origins of the cortical profiles obtained in our analysis, and provide some reassurance of their validity. Further supporting this, the solution revealed here also shows several analogies to the clustering solution of genetic contribution to SA and CT reported by Chen et al. (2013) and might be especially relevant in early development where external environmental effects on these parameters are still relatively small.

The clustering solutions were spatially robust, by and large not affected by age (confirmed by clustering separately only individuals scanned in the top and bottom PMA-range quartiles), nor was it affected by the neonates’ ex utero experience, as the clustering solution for neonates scanned within a week from birth resembled that of the entire sample (data not shown). We also show that the clustering solution is not driven exclusively by age-related changes in the morphometric measures (Supplementary Fig. 3). So, although age did not alter the location of structure of the MSNs, it was associated with their internal coherence. Age was linked to increased regional similarity within the clusters and both increased and decreased regional similarity between clusters (cluster integration and segregation). Though the exact pattern was complex, the summary was that cingulate showed integration with the limbic and insular clusters while segregating from most of the neocortical clusters, perhaps suggesting an advancement towards a more broad representation of paralimbic structures, while the limbic and fronto–temporal clusters showed also age-dependent increase in intrasimilarity.

While our study’s advantages include a large neonatal sample, data acquired and analyzed with optimized protocols for this age group, as well as the use of a number of morphometric measures to characterize the brain, it is not without limitations. In order to fully describe development, longitudinal data is needed. Due to the nature of the surface morphometric measures utilized, we were not able to assess whole-brain connectivity, here excluding subcortical structures, cerebellum, and brain stem. Future work should investigate the structural and functional coupling of neonatal MSNs, as well as their genetic and environmental correlates. The implication of recent work using MSNs is that changes in neurodevelopmental disorders must occur early in development. Using a large sample, our study provides a means to detect meaningful alterations in structural coupling at an early stage in infants with either a known genetic or high likelihood to have a later neurodevelopmental disorder.

To conclude, we present cortical MSNs in the neonatal brain, their association with age, and their community structure, characterizing evolving cortical tissue architecture. Following birth, age is strongly related to edge-strength, in a posterior–anterior gradient reflecting the directionality of axonal and dendritic growth, myelination, and synaptogenesis in this period. Clustering of MSNs into modules demonstrated correspondence to known functional systems and cytoarchitectural atlases and was relatively stable across subjects and over the age range examined. Within these clusters, similarity generally seemed to increase postnatally, implying increased network coherence in this short period. We also find a complex relationship of between-clusters similarity, suggesting some rearrangement in segregation and integration of the network, indicative of cortical maturational processes. Here we provide a template of neonatal structural cortical co-variance profiles. By utilizing both shape and microstructural information, we highlight the complex nature of perinatal cortical development.

## Supplementary Material

supp_bhaa150

## Notes

We would like to thank the infants and parents who contributed their time to this research. We are also thankful to our colleagues from the Newborn Imaging Centre at Evelina London. *Conflict of Interest*. None declared.

## References

[ref1] Alexander-Bloch A, Giedd JN, Bullmore E 2013a Imaging structural co-variance between human brain regions. Nat Rev Neurosci. 14:322–336. 23531697 10.1038/nrn3465 PMC4043276

[ref2] Alexander-Bloch A, Raznahan A, Bullmore E, Giedd J 2013b The convergence of maturational change and structural covariance in human cortical networks. J Neurosci. 33:2889–2899. 23407947 10.1523/JNEUROSCI.3554-12.2013 PMC3711653

[ref3] Alexander-Bloch AF, Shou H, Liu S, Satterthwaite TD, Glahn DC, Shinohara RT, Vandekar SN, Raznahan A 2018 On testing for spatial correspondence between maps of human brain structure and function. Neuroimage. 178:540–551. 29860082 10.1016/j.neuroimage.2018.05.070 PMC6095687

[ref4] Andersson JLR, Skare S, Ashburner J 2003 How to correct susceptibility distortions in spin-echo echo-planar images: application to diffusion tensor imaging. Neuroimage. 20:870–888. 14568458 10.1016/S1053-8119(03)00336-7

[ref5] Arslan S, Ktena SI, Makropoulos A, Robinson EC, Rueckert D, Parisot S 2018 Human brain mapping: a systematic comparison of parcellation methods for the human cerebral cortex. Neuroimage. 170:5–30. 28412442 10.1016/j.neuroimage.2017.04.014

[ref6] Ball G, Aljabar P, Nongena P, Kennea N, Gonzalez-Cinca N, Falconer S, Chew ATM, Harper N, Wurie J, Rutherford MA et al. 2017 Multimodal image analysis of clinical influences on preterm brain development. Ann Neurol. 82:233–246. 28719076 10.1002/ana.24995 PMC5601217

[ref7] Ball G, Srinivasan L, Aljabar P, Counsell SJ, Durighel G, Hajnal JV, Rutherford MA, Edwards AD 2013 Development of cortical microstructure in the preterm human brain. Proc Natl Acad Sci USA. 110:9541–9546. 23696665 10.1073/pnas.1301652110 PMC3677430

[ref8] Batalle D, Hughes EJ, Zhang H, Tournier JD, Tusor N, Aljabar P, Wali L, Alexander DC, Hajnal JV, Nosarti C et al. 2017 Early development of structural networks and the impact of prematurity on brain connectivity. Neuroimage. 149:379–392. 28153637 10.1016/j.neuroimage.2017.01.065 PMC5387181

[ref9] Batalle D, O’Muircheartaigh J, Makropoulos A, Kelly CJ, Dimitrova R, Hughes EJ, Hajnal JV, Zhang H, Alexander DC, Edwards AD et al. 2019 Different patterns of cortical maturation before and after 38 weeks gestational age demonstrated by diffusion MRI in vivo. Neuroimage. 185:764–775. 29802969 10.1016/j.neuroimage.2018.05.046 PMC6299264

[ref10] Benjamini Y, Hochberg Y 1995 Controlling the false discovery rate: a practical and powerful approach to multiple testing. J R Stat Soc Ser B. 57:289–300.

[ref11] Betzel RF, Medaglia JD, Papadopoulos L, Baum GL, Gur R, Gur R, Roalf D, Satterthwaite TD, Bassett DS 2017 The modular organization of human anatomical brain networks: accounting for the cost of wiring. Netw Neurosci. 1:42–68. 30793069 10.1162/NETN_a_00002 PMC6372290

[ref12] Bordier C, Nicolini C, Bifone A 2017 Graph analysis and modularity of brain functional connectivity networks: searching for the optimal threshold. Front Neurosci. 11:441. 28824364 10.3389/fnins.2017.00441 PMC5540956

[ref13] Bozek J, Makropoulos A, Schuh A, Fitzgibbon S, Wright R, Glasser MF, Coalson TS, O’Muircheartaigh J, Hutter J, Price AN et al. 2018 Construction of a neonatal cortical surface atlas using multimodal surface matching in the developing human connectome project. Neuroimage. 179:11–29. 29890325 10.1016/j.neuroimage.2018.06.018 PMC6783315

[ref14] Cao M, Huang H, Peng Y, Dong Q, He Y 2016 Toward developmental connectomics of the human brain. Front Neuroanat. 10:25. 27064378 10.3389/fnana.2016.00025 PMC4814555

[ref15] Chen C-H, Fiecas M, Gutiérrez ED, Panizzon MS, Eyler LT, Vuoksimaa E, Thompson WK, Fennema-Notestine C, Hagler DJ, Jernigan TL et al. 2013 Genetic topography of brain morphology. Proc Natl Acad Sci USA. 110:17089–17094. 24082094 10.1073/pnas.1308091110 PMC3801007

[ref16] Christiaens D, Cordero-Grande L, Hutter J, Price AN, Deprez M, Hajnal JV, Tournier J-D 2019a Learning compact q-space representations for multi-shell diffusion-weighted MRI. IEEE Trans Med Imaging. 38:834–843. 30296214 10.1109/TMI.2018.2873736

[ref17] Christiaens D, Cordero-Grande L, Pietsch M, Hutter J, Price AN, Hughes EJ, Vecchiato K, Deprez M, Edwards AD, Hajnal JV, et al. 2019b Scattered slice SHARD reconstruction for motion correction in multi-shell diffusion MRI of the neonatal brain. arXiv. 1905.02996. 10.1016/j.neuroimage.2020.117437 PMC7779423 33068713

[ref18] Cordero-Grande L, Hughes EJ, Hutter J, Price AN, Hajnal JV 2018 Three-dimensional motion corrected sensitivity encoding reconstruction for multi-shot multi-slice MRI: application to neonatal brain imaging. Magn Reson Med. 79:1365–1376. 28626962 10.1002/mrm.26796 PMC5811842

[ref19] Cordero-Grande L, Teixeira RPAG, Hughes EJ, Hutter J, Price AN, Hajnal JV 2016 Sensitivity encoding for aligned multishot magnetic resonance reconstruction. IEEE Trans Comput Imaging. 2:266–280.

[ref20] Dean DC, Planalp EM, Wooten W, Adluru N, Kecskemeti SR, Frye C, Schmidt CK, Schmidt NL, Styner MA, Goldsmith HH et al. 2017 Mapping white matter microstructure in the one month human brain. Sci Rep. 7:9759. 28852074 10.1038/s41598-017-09915-6 PMC5575288

[ref21] Di Martino A, Fair DA, Kelly C, Satterthwaite TD, Castellanos FX, Thomason ME, Craddock RC, Luna B, Leventhal BL, Zuo X-N et al. 2014 Unraveling the miswired connectome: a developmental perspective. Neuron. 83:1335–1353. 25233316 10.1016/j.neuron.2014.08.050 PMC4169187

[ref22] Evans AC 2013 Networks of anatomical covariance. Neuroimage. 80:489–504. 23711536 10.1016/j.neuroimage.2013.05.054

[ref23] Fan Y, Shi F, Smith JK, Lin W, Gilmore JH, Shen D 2011 Brain anatomical networks in early human brain development. Neuroimage. 54:1862–1871. 20650319 10.1016/j.neuroimage.2010.07.025 PMC3023885

[ref24] Feis D-L, Brodersen KH, von Cramon DY, Luders E, Tittgemeyer M 2013 Decoding gender dimorphism of the human brain using multimodal anatomical and diffusion MRI data. Neuroimage. 70:250–257. 23298750 10.1016/j.neuroimage.2012.12.068

[ref25] Flechsig of Leipsic P 1901 Developmental (Myelogenetic) localisation of the cerebral cortex in the human subject. Lancet. 158:1027–1030.

[ref26] Frey BJ, Dueck D 2007 Clustering by passing messages between data points. Science (80-). 315:972–976. 10.1126/science.1136800 17218491

[ref27] Galdi P, Blesa M, Stoye DQ, Sullivan G, Lamb GJ, Quigley AJ, Thrippleton MJ, Bastin ME, Boardman JP 2020 Neonatal morphometric similarity mapping for predicting brain age and characterizing neuroanatomic variation associated with preterm birth. NeuroImage Clin. 102195. 32044713 10.1016/j.nicl.2020.102195 PMC7016043

[ref28] Geng X, Li G, Lu Z, Gao W, Wang L, Shen D, Zhu H, Gilmore JH 2017 Structural and maturational covariance in early childhood brain development. Cereb Cortex. 27:1795–1807. 26874184 10.1093/cercor/bhw022 PMC6059236

[ref29] Gilmore JH, Shi F, Woolson SL, Knickmeyer RC, Short SJ, Lin W, Zhu H, Hamer RM, Styner M, Shen D 2012 Longitudinal development of cortical and subcortical gray matter from birth to 2 years. Cereb Cortex. 22:2478–2485. 22109543 10.1093/cercor/bhr327 PMC3464410

[ref30] Gilmore JH, Lin W, Prastawa MW, Looney CB, Vetsa YSK, Knickmeyer RC, Evans DD, Smith JK, Hamer RM, Lieberman JA et al. 2007 Regional gray matter growth, sexual dimorphism, and cerebral asymmetry in the neonatal brain. J Neurosci. 27:1255–1260. 17287499 10.1523/JNEUROSCI.3339-06.2007 PMC2886661

[ref31] Glasser MF, Van Essen DC 2011 Mapping human cortical areas in vivo based on myelin content as revealed by T1- and T2-weighted MRI. J Neurosci. 31:11597–11616. 21832190 10.1523/JNEUROSCI.2180-11.2011 PMC3167149

[ref32] Goulas A, Werner RR, Beul SF, Saering D, van den Heuvel M, Triarhou LC, Hilgetag CC, Säring D, Van Den Heuvel M, Triarhou LC et al. 2016 Cytoarchitectonic similarity is a wiring principle of the human connectome. bioRxiv. 068254.

[ref33] Guerrero JM, Adluru N, Bendlin BB, Goldsmith HH, Schaefer SM, Davidson RJ, Kecskemeti SR, Zhang H, Alexander AL 2019 Optimizing the intrinsic parallel diffusivity in NODDI: an extensive empirical evaluation. PLoS One. 14:e0217118. 31553719 10.1371/journal.pone.0217118 PMC6760776

[ref34] Hughes EJ, Winchman T, Padormo F, Teixeira R, Wurie J, Sharma M, Fox M, Hutter J, Cordero-Grande L, Price AN et al. 2017 A dedicated neonatal brain imaging system. Magn Reson Med. 78:794–804. 27643791 10.1002/mrm.26462 PMC5516134

[ref35] Huttenlocher PR 1979 Synaptic density in human frontal cortex - developmental changes and effects of aging. Brain Res. 163:195–205. 427544 10.1016/0006-8993(79)90349-4

[ref36] Huttenlocher PR 1990 Morphometric study of human cerebral cortex development. Neuropsychologia. 28:517–527. 2203993 10.1016/0028-3932(90)90031-i

[ref37] Huttenlocher PR, Dabholkar AS 1997 Regional differences in synaptogenesis in human cerebral cortex. J Comp Neurol. 387:167–178. 9336221 10.1002/(sici)1096-9861(19971020)387:2<167::aid-cne1>3.0.co;2-z

[ref38] Hutter J, Tournier JD, Price AN, Cordero-Grande L, Hughes EJ, Malik S, Steinweg J, Bastiani M, Sotiropoulos SN, Jbabdi S et al. 2018 Time-efficient and flexible design of optimized multishell HARDI diffusion. Magn Reson Med. 79:1276–1292. 28557055 10.1002/mrm.26765 PMC5811841

[ref39] Kellner E, Dhital B, Kiselev VG, Reisert M 2016 Gibbs-ringing artifact removal based on local subvoxel-shifts. Magn Reson Med. 76:1574–1581. 26745823 10.1002/mrm.26054

[ref40] Knickmeyer RC, Wang J, Zhu H, Geng X, Woolson S, Hamer RM, Konneker T, Styner M, Gilmore JH 2014 Impact of sex and gonadal steroids on neonatal brain structure. Cereb Cortex. 24:2721–2731. 23689636 10.1093/cercor/bht125 PMC4153808

[ref41] Kuklisova-Murgasova M, Quaghebeur G, Rutherford MA, Hajnal JV, Schnabel JA 2012 Reconstruction of fetal brain MRI with intensity matching and complete outlier removal. Med Image Anal. 16:1550–1564. 22939612 10.1016/j.media.2012.07.004 PMC4067058

[ref42] Lebenberg J, Mangin J-F, Thirion B, Poupon C, Hertz-Pannier L, Leroy F, Adibpour P, Dehaene-Lambertz G, Dubois J 2019 Mapping the asynchrony of cortical maturation in the infant brain: a MRI multi-parametric clustering approach. Neuroimage. 185:641–653. 30017787 10.1016/j.neuroimage.2018.07.022

[ref43] Li G, Nie J, Wang L, Shi F, Lin W, Gilmore JH, Shen D 2013 Mapping region-specific longitudinal cortical surface expansion from birth to 2 years of age. Cereb Cortex. 23:2724–2733. 22923087 10.1093/cercor/bhs265 PMC3792744

[ref44] Li G, Wang L, Shi F, Gilmore JH, Lin W, Shen D 2015 Construction of 4D high-definition cortical surface atlases of infants: methods and applications. Med Image Anal. 25:22–36. 25980388 10.1016/j.media.2015.04.005 PMC4540689

[ref45] Li W, Yang C, Shi F, Wu S, Wang Q, Nie Y, Zhang X 2017 Construction of individual morphological brain networks with multiple morphometric features. Front Neuroanat. 11:34. 28487638 10.3389/fnana.2017.00034 PMC5403938

[ref46] Lyall AE, Shi F, Geng X, Woolson S, Li G, Wang L, Hamer RM, Shen D, Gilmore JH 2015 Dynamic development of regional cortical thickness and surface area in early childhood. Cereb Cortex. 25:2204–2212. 24591525 10.1093/cercor/bhu027 PMC4506327

[ref47] Makropoulos A, Robinson EC, Schuh A, Wright R, Fitzgibbon S, Bozek J, Counsell SJ, Steinweg J, Vecchiato K, Passerat-Palmbach J et al. 2018 The developing human connectome project: a minimal processing pipeline for neonatal cortical surface reconstruction. Neuroimage. 173:88–112. 29409960 10.1101/125526 PMC6783314

[ref48] Meilă M 2007 Comparing clusterings—an information based distance. J Multivar Anal. 98:873–895.

[ref49] Morgan SE, White SR, Bullmore ET, Vértes PE 2018 A network neuroscience approach to typical and atypical brain development. Biol Psychiatry Cogn Neurosci Neuroimaging. 3:754–766. 29703679 10.1016/j.bpsc.2018.03.003 PMC6986924

[ref50] Morgan SE, Seidlitz J, Whitaker KJ, Romero-Garcia R, Clifton NE, Scarpazza C, van Amelsvoort T, Marcelis M, van Os J, Donohoe G et al. 2019 Cortical patterning of abnormal morphometric similarity in psychosis is associated with brain expression of schizophrenia-related genes. Proc Natl Acad Sci USA. 116:9604–9609. 31004051 10.1073/pnas.1820754116 PMC6511038

[ref51] Nie J, Li G, Shen D 2013 Development of cortical anatomical properties from early childhood to early adulthood. Neuroimage. 76:216–224. 23523806 10.1016/j.neuroimage.2013.03.021 PMC3752662

[ref52] Nie J, Li G, Wang L, Shi F, Lin W, Gilmore JH, Shen D 2014 Longitudinal development of cortical thickness, folding, and fiber density networks in the first 2 years of life. Hum Brain Mapp. 35:3726–3737. 24375724 10.1002/hbm.22432 PMC4065646

[ref53] Panizzon MS, Fennema-Notestine C, Eyler LT, Jernigan TL, Prom-Wormley E, Neale M, Jacobson K, Lyons MJ, Grant MD, Franz CE et al. 2009 Distinct genetic influences on cortical surface area and cortical thickness. Cereb Cortex. 19:2728–2735. 19299253 10.1093/cercor/bhp026 PMC2758684

[ref54] Paredes MF, James D, Gil-Perotin S, Kim H, Cotter JA, Ng C, Sandoval K, Rowitch DH, Xu D, McQuillen PS et al. 2016 Extensive migration of young neurons into the infant human frontal lobe. Science (80-). 354:aaf7073. 10.1126/science.aaf7073 PMC5436574 27846470

[ref55] Petanjek Z, Judaš M, Šimić G, Rašin MR, Uylings HBM, Rakic P, Kostović I 2011 Extraordinary neoteny of synaptic spines in the human prefrontal cortex. Proc Natl Acad Sci USA. 108:13281–13286. 21788513 10.1073/pnas.1105108108 PMC3156171

[ref56] Rakic P 1988 Specification of cerebral cortical areas. Science. 241:170–176. 3291116 10.1126/science.3291116

[ref57] Reichart R, Rappoport A 2009 The NVI clustering evaluation measure In: Proceedings of the Thirteenth Conference on Computational Natural Language Learning, 165–173.

[ref58] Robinson EC, Garcia K, Glasser MF, Chen Z, Coalson TS, Makropoulos A, Bozek J, Wright R, Schuh A, Webster M et al. 2018 Multimodal surface matching with higher-order smoothness constraints. Neuroimage. 167:453–465. 29100940 10.1016/j.neuroimage.2017.10.037 PMC5991912

[ref59] Robinson EC, Jbabdi S, Andersson J, Smith S, Glasser MF, Van Essen DC, Burgess G, Harms MP, Barch DM, Jenkinson M 2013 Multimodal surface matching: Fast and generalisable cortical registration using discrete optimisation In: Gee JC, Joshi S, Pohl KM, Wells WMZL, editors. Information processing in medical imaging. Lecture notes in computer science. Berlin, Heidelberg: Springer, pp. 475–486. 10.1007/978-3-642-38868-2_40 24683992

[ref60] Robinson EC, Jbabdi S, Glasser MF, Andersson J, Burgess GC, Harms MP, Smith SM, Van Essen DC, Jenkinson M 2014 MSM: a new flexible framework for multimodal surface matching. Neuroimage. 100:414–426. 24939340 10.1016/j.neuroimage.2014.05.069 PMC4190319

[ref61] Romero-Garcia R, Whitaker KJ, Váša F, Seidlitz J, Shinn M, Fonagy P, Dolan RJ, Jones PB, Goodyer IM, Bullmore ET et al. 2018 Structural covariance networks are coupled to expression of genes enriched in supragranular layers of the human cortex. Neuroimage. 171:256–267. 29274746 10.1016/j.neuroimage.2017.12.060 PMC5883331

[ref62] Seghier ML, Price CJ 2018 Interpreting and utilising intersubject variability in brain function. Trends Cogn Sci. 22:517–530. 29609894 10.1016/j.tics.2018.03.003 PMC5962820

[ref63] Seidlitz J, Váša F, Shinn M, Romero-Garcia R, Whitaker KJ, Vértes PE, Wagstyl K, Kirkpatrick Reardon P, Clasen L et al. 2018 Morphometric similarity networks detect microscale cortical organization and predict inter-individual cognitive variation. Neuron. 97:231–247.e7. 29276055 10.1016/j.neuron.2017.11.039 PMC5763517

[ref64] Seidlitz J, Nadig A, Liu S, Bethlehem RAI, Vértes PE, Morgan SE, Váša F, Romero-Garcia R, Lalonde FM, Clasen LS et al. 2019 Transcriptomic and cellular decoding of regional brain vulnerability to neurodevelopmental disorders. bioRxiv. 573279.

[ref65] Shi F, Yap PT, Gao W, Lin W, Gilmore JH, Shen D 2012 Altered structural connectivity in neonates at genetic risk for schizophrenia: a combined study using morphological and white matter networks. Neuroimage. 62:1622–1633. 22613620 10.1016/j.neuroimage.2012.05.026 PMC3408572

[ref66] Sporns O, Betzel RF 2016 Modular brain networks. Annu Rev Psychol. 67:613–640. 26393868 10.1146/annurev-psych-122414-033634 PMC4782188

[ref67] Storey JD 2002 A direct approach to false discovery rates. J R Stat Soc Ser B (Statistical Methodol). 64:479–498.

[ref68] Storey JD, Tibshirani R 2003 Statistical significance for genome-wide studies. Proc Natl Acad Sci USA. 100:9440–9445. 12883005 10.1073/pnas.1530509100 PMC170937

[ref69] Teffer K, Semendeferi K 2012 Human prefrontal cortex: evolution, development, and pathology. Prog Brain Res. 195:191–218. 22230628 10.1016/B978-0-444-53860-4.00009-X

[ref70] Tournier JD, Hughes E, Tusor N, Sotiropoulos SN, Jbabdi S, Andersson J, Rueckert D, Edwards AD, Hajnal JV 2015 Data-driven optimisation of multi-shell HARDI In: Proceedings of the 23rd Annual Meeting of ISMRM. Toronto, Canada, p. 2897.

[ref71] Tournier JD, Smith R, Raffelt D, Tabbara R, Dhollander T, Pietsch M, Christiaens D, Jeurissen B, Yeh CH, Connelly A 2019 MRtrix3: a fast, flexible and open software framework for medical image processing and visualisation. Neuroimage. 202:116137. 31473352 10.1016/j.neuroimage.2019.116137

[ref72] van den Heuvel MP, Kersbergen KJ, de Reus MA, Keunen K, Kahn RS, Groenendaal F, de Vries LS, Benders MJNL 2015 The neonatal connectome during preterm brain development. Cereb Cortex. 25:3000–3013. 24833018 10.1093/cercor/bhu095 PMC4537441

[ref73] Veraart J, Novikov DS, Christiaens D, Ades-aron B, Sijbers J, Fieremans E 2016 Denoising of diffusion MRI using random matrix theory. Neuroimage. 142:394–406. 27523449 10.1016/j.neuroimage.2016.08.016 PMC5159209

[ref74] von Economo C, Koskinas G 1925 Die Cytoarchitektonik der Hirnrinde des erwachsenen Menschen. Berlin: Springer.

[ref75] Whitaker KJ, Vértes PE, Romero-Garciaa R, Váša F, Moutoussis M, Prabhu G, Weiskopf N, Callaghan MF, Wagstyl K, Rittman T et al. 2016 Adolescence is associated with genomically patterned consolidation of the hubs of the human brain connectome. Proc Natl Acad Sci U S A. 113:9105–9110. 27457931 10.1073/pnas.1601745113 PMC4987797

[ref76] Yee Y, Fernandes DJ, French L, Ellegood J, Cahill LS, Vousden DA, Spencer Noakes L, Scholz J, van Eede MC, Nieman BJ et al. 2018 Structural covariance of brain region volumes is associated with both structural connectivity and transcriptomic similarity. Neuroimage. 179:357–372. 29782994 10.1016/j.neuroimage.2018.05.028

[ref77] Zhang H, Schneider T, Wheeler-Kingshott CA, Alexander DC 2012 NODDI: practical in vivo neurite orientation dispersion and density imaging of the human brain. Neuroimage. 61:1000–1016. 22484410 10.1016/j.neuroimage.2012.03.072

[ref78] Zhao T, Xu Y, He Y 2019 Graph theoretical modeling of baby brain networks. Neuroimage. 185:711–727. 29906633 10.1016/j.neuroimage.2018.06.038

[ref79] Zielinski BA, Gennatas ED, Zhou J, Seeley WW 2010 Network-level structural covariance in the developing brain. Proc Natl Acad Sci USA. 107:18191–18196. 20921389 10.1073/pnas.1003109107 PMC2964249

[ref80] Zuo Z, Ran S, Wang Y, Li C, Han Q, Tang Q, Qu W, Li H 2018 Altered structural covariance among the dorsolateral prefrontal cortex and amygdala in treatment-Naïve patients with major depressive disorder. Front Psych. 9:323. 10.3389/fpsyt.2018.00323 PMC6062642 30079037

